# Understanding Wildlife Crime from Eco-Existential and African Perspectives: A Psycho-Philosophical Investigation

**DOI:** 10.3390/ijerph182111675

**Published:** 2021-11-07

**Authors:** Claude-Hélène Mayer

**Affiliations:** Department of Industrial Psychology and People Management, University of Johannesburg, Johannesburg 2006, South Africa; claudemayer@gmx.net

**Keywords:** wildlife crime, conservation, existential psychology, eco-existentialism, African philosophy, existential givens

## Abstract

Wildlife crime has huge consequences regarding global environmental changes to animals, plants and the entire ecosystem. Combatting wildlife crime effectively requires a deep understanding of human–wildlife interactions and an analysis of the influencing factors. Conservation and green criminology are important in reducing wildlife crime, protecting wildlife and the ecosystem and informing policy-makers about best practices and strategies. However, the past years have shown that wildlife crime is not easy to combat and it is argued in this article that there are underlying existential “givens” and culture-specific aspects that need to be investigated to understand why wildlife crime is still on the rise. This theoretical article explores (eco-)existential perspectives, Greening’s four givens and selected African philosophical concepts, aiming to understand the complexities behind the prevalence of wildlife crime within global and African contexts.

## 1. Introduction 

Human–animal studies have gained interest during recent years, focusing on exploring the relationships between humans and animals from different theoretical and methodological perspectives [1,2,3]. Most of the studies have focused on pets and the use of animals for entertainment and work-related aspects, while most of the authors conducting these studies are based in the US. Only recently has the topic gained increased popularity in Europe [4]. In African contexts, research on human–animal studies is extremely rare, and studies exploring human–wildlife interactions and relationships from psychological, social and criminological disciplinary perspectives are still underrepresented [5,6,7]. However, the topic of wildlife crime has gained momentum, since reductions in biodiversity and the extinction of several wildlife species have evoked concern and research in this area [8,9,10]. Wildlife crime has huge consequences regarding global environmental changes to animals, plants and the entire ecosystem [11]. Wildlife crime includes “environment-related crimes that involve the illegal trade, smuggling, poaching, capture or collection of endangered species, protected wildlife (including animal and plants that are subject to harvest quotas and regulated permits) derivatives or products thereof” [8]. Wildlife crime negatively impacts on the financial, economic and sociocultural sustainability of the ecosystem and is caused by human beings [10].

Combatting wildlife crime effectively requires a deep understanding of human–wildlife interactions and an analysis of the contextual influencing factors [9]. Conservation science and green criminology are important in reducing wildlife crime, protecting the ecosystem and wildlife and informing policy-makers about best practices and strategies—eradicating failures, errors and mistakes—in order to finally combat crime effectively at global, regional and local levels [6,12]. Researchers from the Global South have called for a specific southern green criminology perspective recently, focusing in particular on aspects of (de-)colonialization; the epistemological contributions of the marginalised, impoverished and oppressed; and the particularities of the contexts of the Global South [13,14,15]. However, often studies on combatting wildlife crime in the Global South are reduced to specific investigation tools, strategies, policy discourses and strategic management approaches, as well as Western scientific perspectives [6]. 

Goyes and Bennett [16] have recently called for a deeper integration of wildlife crime investigation and conservation sciences, while Plous [17] already mentioned in the early 1990s that the psychological mechanisms of the human use of animals need to be taken into consideration when dealing with behavioural patterns of human–animal interactions. This article follows these calls and highlights the fact that (1) psychological and philosophical implications from eco-existential perspectives on wildlife crime and (2) Southern African sociocultural perspectives are underexplored in the literature. 

**Figure 1:**
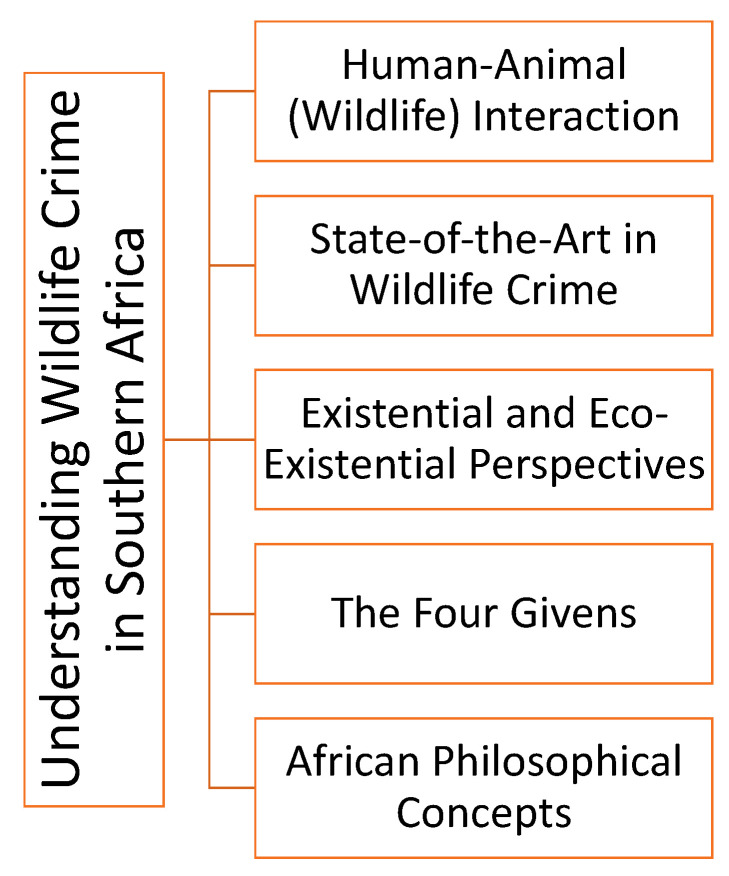
Understanding wildlife crime.

The aim of this article is to understand selected existentialist and African perspectives on human–animal interactions and wildlife crime to create a deeper understanding of the underlying psychological and philosophical implications of wildlife crime and why it seems to be extremely difficult to combat it on global levels (Figure 1).

This article uses Greening’s [1] four dialectical existential givens—*life/death*, *meaning/absurdity*, *freedom/determinism* and *community/isolation*—as a guiding framework to analyse the psychological and philosophical ideas underlying the continuing and even accelerating rates of wildlife crime. The discourse on human–animal interactions from existential and African perspectives aims to improve human–animal interactions and is anchored in eco-existential and African thoughts.

In the following sections, the context will be presented, the state-of-the-art regarding wildlife and wildlife crime will be discussed and eco-existential and African perspectives will be introduced. Finally, based on the new theoretical insights, practical approaches to addressing wildlife crime will be presented.

## 2. The Contextual Background 

COVID-19, as a global crisis, has caused suffering and pain for individuals and humankind, thereby causing momentum to rethink habitual patterns of behaviour and human–animal interactions [18]. Since COVID-19 was first reported as having spread from the Huanan seafood wholesale market in Wuhan city, in the Hubei province in China [19], attention has been drawn to the question of the origin of the virus. Andersen et al. [20] pointed out that the species originated from *Phinolophus affinis* (horseshoe bats) with zoonotic spillover in a *Manis javanica* intermediary host (Malayan pangolins). Due to the problematic transmission of the virus from (wildlife) animals to humans in the context of illegal wildlife markets and wildlife trade, the pandemic has evoked critical voices to speak up against wildlife crime and to promote more careful and caring human–animal relationships with an ecocentric perspective [21,22].

One might say that the pandemic has brought about this “urgent experience” [23], of the rapid changes in lifestyle, suffering and pain to humans to reflect in particular on their behavioural patterns and relationships with nature, animals and wildlife. It is argued here that the urgent experience of the pandemic does not necessarily bring about changes in patterns of behaviour or in human–animal relationships as such; it rather evokes in-depth reflection and changes in deep and complex mindsets and philosophical assumptions to comprehend the patterns of behaviour on deeper levels. Based on these deeper reflections, new behavioural patterns might emerge. For example, China has banned further sales of wild animals for human consumption. However, according to the Humane Society International (21, p. 2), “the terms of the decision remain ambiguous”. Global authorities have called for new international agreements to prevent and stop wildlife crime as an essential step to prevent the spread of current and future zoonoses (animal originated infectious agents being spread to a human), which are said to be the cause of HIV, AIDS, Ebola, SARS, MERS and COVID-19 [24]. 

Drawing on existential–humanistic theory, it is suggested that dialectical worldviews and paradoxes—from an existential–humanistic perspective—can be transcended by confronting positive and negative aspects that are simplistically overemphasised [23]. Through the COVID-19 experience, it is assumed that consciousness, caring and collective co-creation can be enhanced [23], if explored from an in-depth, critical and reflective mindset. That means that human–animal relationships, and specifically wildlife crime as an example for negative human–animal relationships, need to be understood through in-depth reflections to comprehend the root causes rather than to act upon applied assumptions. 

### 2.1. Human–Animal (Wildlife) Interactions and Wildlife Crime in Southern Africa

The interest in and studies on wild animals expanded after the increase in motorisation and the development of national parks and environmentally protected areas in which humans started observing wild animals on safaris and hunting them [2]. From an ethical perspective, wildlife hunting is viewed as an elite sport that is as unethical, since it is based on the joy of killing [25]. For example, the latest reports have shown that the South African government aims to stop wildlife hunting of lions in fenced areas due to the practice being unethical [26]. 

Often wildlife and human–wildlife interactions have been discussed with regard to wild animals’ dependence on human beings [27]. Wild animals are dependent on the protection of their land and territory through humans, but also depend on humans to protect their lives [2]. At the same time, people compete with wildlife for food and resources, which has led to specific impacts on human–animal interactions, resulting in co-existence as well as conflict [28]. The two main discourses on human–wildlife interactions focus on the limited agency of wildlife due to human impacts and the overwhelming expansion of wildlife crime. Wildlife crime covers several different forms of crime, including trafficking; poaching or consuming of wildlife [29]; and trading of wildlife articles, such as skin, leather, souvenirs and living animals and pets [30]. During the past decades, one of the major global challenges has been tackling the well-equipped transnational organised crime syndicates involved in wildlife crime, which are extremely successful due to frequent corruption, prosecution delays and poor evidence-handling [31]. Internationally, wildlife crime generates some of the greatest illicit revenue streams, particular from illegal wildlife markets [6,32,33]. The largest shares of income from wildlife crime are generated at the retail level, although no concrete numbers exist regarding the value of the wildlife products traded around the world. Wildlife crime causes huge damages at environmental, social, cultural and financial levels [34,35], so much so that it even threatens human existence in the present and future [36]. Over many years, the international community has aimed to combat wildlife crime on micro, meso and macro levels [37], but has failed terribly in many respects [12]. Why is that so?

Managing wildlife crime globally has mainly been attempted at the policy level from Western perspectives [8], in terms of developing methods, investigative tools, governments and international networks to combat wildlife crime syndicates [6,38,39,40]. However, wildlife crime within the context of human–animal interactions has hardly been studied from eco-existential, African policy and investigative perspectives [6,12,41], and even less so from philosophical and psychological perspectives, which might clarify the underlying causes.

Often research on wildlife crime is anchored in Western theories and practices to deal with complex structures, networks and mindsets in the Global South. Western policy-makers do not seem to be serious about the issues of Southern African wildlife crime [42]. Combatting wildlife crime requires one to take a very practical approach by addressing the underlying causes, such as poverty, community-driven wildlife crime, poor governance of African states, local poaching activities, socio-historical problems of cultural and ethnic groups and inequality, factors that provide a solid base for the unconscious expansion of wildlife crime via oppressed groups within African states, e.g., as supported by apartheid in South Africa [12]. Further, the occurrence of wildlife crime is also ascribed to the lack of wildlife laws and environmental legislation [43]. Finally, wildlife crime combat is often neglected because of the use of conservationism as a political tool to promote selected socio-cultural groups and due to the frequently experienced clashes over traditional land owner rights in conservation areas [6]. It is argued here that wildlife crime combat has not only failed because of the causes presented above, but also because of underlying philosophical and psychological mindsets that have been left unaddressed.

### 2.2. Philosophical and Psychological Approaches to Wildlife Crime

As emphasised in the past, wildlife crime has mainly been addressed using practical tools and interventions to combat it, with practical failures occurring in the process. [44,45]. It is argued here that this is not enough. On the one hand, wildlife crime and combatting such crime need to be approached from psychological and philosophical stances that take ethical and moral aspects and their implications for human–human and human–animal relationships into consideration [46,47], which can then impact the collaboration between legal and illegal spheres [48]. On the other hand, combatting such crime requires culture-specific views to be taken into consideration, which may play a role in building new perspectives on human–animal interactions and help wildlife crime to be successfully combatted from a culture-specific and eco-centralised perspective [12].

Within the context of the COVID-19 outbreak, it has been previously pointed out by the South African wildlife conservationist and philosopher Adam Cruise [49] that humanity needs to take a second look at its relationship with human nature and to reset the commercialization of nature and animals in farming, wildlife management and diets. As such, anthropocentrism has been criticised as an egotistical view of ecosystems rather than a holistic and nature-bound view of the current situation [50]. Values, such as self-love, self-respect and consciousness in humans, are supportive in moving from anthropocentric, human-centred approaches towards eco-centric approaches [50,51]. There is an ongoing debate regarding the impacts of both approaches on conservation. Some authors have stated that anthropocentrism is benign for conservation [52], while others have stated that ecocentrism is inevitable in biodiversity conservation [53]. Manolopoulos [54] presented an earth- and eco-centred leadership approach towards nature, which involved several leadership stages arguing for ecological equality in the world. With regard to the protection of wildlife and the acceptance of animals and wildlife as equal beings and ecosystem engineers, Tague [27] called for the acknowledgement of ape personhood and ape ethics, radically emphasizing that humans should accept wildlife and animals as equal, referring to apes an example.

### 2.3. Existential and Eco-Existential Perspectives

Existentialism proposes that the most important fundamental of human life is that human beings exist, live and feel [46]. In human–animal studies, ethical questions have been highlighted during the past decade, which often take an ecological, holistic and even spiritual view of human–animal relationships that is anchored in ideas of ecological and nature-related philosophy [2]. These discourses on ethical and philosophical issues in human–animal interactions are anchored in aspects of the phylogenetic inheritance of humans and animals, the historical influence of the development of humans, the role of creativity and the necessity of respecting the diversity of living beings through culture and being alongside nature [55,56,57].

Eco-existential perspectives in human animal studies go back to psychologists and psychiatrists [58,59,60] and are founded in (existential) European philosophies of the 19th and 20th centuries [61,62]. At the centre of eco-existential perspectives stands the idea that the relationships humans build are extremely important for their mental health and well-being [63,64]. Further, researchers and philosophers have pointed out that the relationships between humans and nature have an evolved inclination. The contact between humans and nature is restorative and additive in its capacity, depending on the perceptions of individuals relating to nature and biophilia [63,65]. Sartre [62], for example, highlights that human beings are born as “nothing” and only become who they are through their choices and actions. These choices are, according to Fromm [59], driven by an inherent human love for nature and all that is living. Wilson [60] takes it even further and proposes the idea that humankind’s connection to nature, beauty and diversity is a genetic component. This concept of biophilia, the love for nature, has often been discussed, and it has been emphasised that biophilia, in relation to animals, is expressed through humankind’s preferences for creating animal metaphors and symbols about nature in language [66]. It has been argued that humankind’s drive towards technology has turned people away from their connection with nature [46,67], leading to an attenuation in humankind’s wish to connect with nature. This might result in a loss of meaning of and respect for nature and its diversity [60] and might lead to the destruction of nature and extinction of animals. Humankind’s connection to nature in terms of emotional bonding and connection to animals is, therefore, viewed as the key to appreciating nature and to reducing natural destruction and animal extinction [68] on the one hand and improving mental health and well-being on the other [64,69]. 

### 2.4. Greening’s Four Existential Givens

During COVID-19, Greening’s [1] four dialectical existential givens have become a focus of attention, which has confronted humanity with an unprecedented global crisis [23,70]. Before Greening [1], the existential therapist Yalom [71] emphasised that in existential therapy, all problems might be reduced to four issues: death, meaninglessness, isolation, and freedom. Kaptelinin [46] argues that insights into issues that existentially address human life and human existence are extremely relevant in developing new existential frameworks of how to deal with the existential issues.

According to Greening [1], life/death, community/isolation, freedom/determinism and meaning/absurdity are four existential givens in the context of the basic challenges of existential human psychology with regard to the human condition. The four existential givens are defined by Greening [1] as follows:

1. Life (and death). We are alive but we will die, and we live a world that both supports and negates life;
2. Meaning (and absurdity). We have a conscious capacity and desire for meaning, but we live in a confusing and sometimes chaotic world that offers many meaning systems and also denies meaning;
3. Freedom (and determinism). We are free and determined, and we live in a world that allows and constricts our freedom;
4. Community (and aloneness). Human desire and capacity for authentic relatedness are countered by inauthenticity, alienation and loneliness.

Eco-existential psychotherapy was introduced by Rank [72] and Tillich [73,74], as well as May [75], Yalom [71] and Frankl [76] during the 20th Century [77], highlighting the fundamental belief that all people experience intrapsychic conflict. This conflict is viewed as inherent to human nature—therefore called human-given—and is anchored in deep feelings of fear and anxiety, which are related to these givens. Existential psychotherapy works towards creating a healthy balance between dealing with the four existential givens, acting to balance awareness about them and coping with them. Passmore and Howell [63] have argued that the well-being of an individual is always connected to the well-being of the larger world. Addressing human–animal interactions and the negative consequences, such as wildlife crime, can increase awareness, meaningfulness, self-respect and responsible decision-making [78]. 

A confrontation with the four givens can bring about new insights, as well as individual development and healing [71]. Humans feel mentally healthy when they can respond to these four existential givens in an adequate way, namely through acceptance and creativity [1]. In this article, the four existential givens will be reflected in the context of human–animal interactions with special regard to African philosophy and wildlife crime.

### 2.5. Life and Death 

In existentialism, it is believed that life is a path to death; it is inherent in nature, and some scholars believe that only humans who confront death can live an authentic life [79]. Tillich [74] emphasises that death is the unknown in life and by its very nature cannot be known.

In African philosophy, life, death and the afterlife are relative similarly defined across different African cultural and ethnic groups [80]. The concept of life includes the ideas that God is the creator of life and the universe; ancestors have an influence in life; and life is “a communal affair” that is defined through interrelationships with God, other humans, ancestors and land [80]. Okolie [81] points out that life in the African perspective is strongly connected to the construction of meaning in life, which again is bound to the idea of living to become a person. To become a person, African ethics point to establishing harmonious relationships of identity and solidarity with the broader aim of helping humans flourish in the world [81].

Death in African contexts is usually not talked about openly [82]. It might be seen as a calling from God who decides that the mission and purpose of life of an individual is fulfilled [83]. However, Mbiti [84] does not ascribe death to a godly force, but rather as an inevitable event caused by the idea that time moves. Further, life is not viewed as ending with death; death is rather a transition to another realm. In this realm, individuals aim become ancestors and their death is viewed as a communitarian event, not an individual transition. To become an ancestor, an individual needs to go through a natural death after having spent a meaningful life [85]. This is because the dead possess supernatural powers and can influence those in the living world [82]. Life and death are viewed as cyclical and the dead are alive in another world and may even reincarnate into this world [82] (Table 1). 

Focusing on life and death in the context of existentialism and African philosophy, existentialists often discuss the nature of killing, particularly with regard to suicide, but also euthanasia and abortion [86]; however, the act of killing animals is hardly taken into consideration. The killing of wild animals for trophies has been described as an existentialist crisis. It has been described as not only a conservationist concern, but also as a strong misalignment with fundamental values in societies. One study [87] highlighted that Western and African moral attitudes and ethics with regard to animal and plant ethics differ fundamentally and that religious and philosophical perceptions and attitudes remain absolutely anthropocentric in African contexts. This means that African traditional thought—although anthropocentric concepts such as *Ubuntu* (humanness) and *Ukama* (relationality) have aimed at including ethical foundations for the improvement of interrelationships between humans and animals—remains exploitative and oppressive regarding human–animal interactions [88]. African philosophers, such as Okpoko [3], have called for an African-centred eco-philosophy that emphasises the reconnection of society with nature. This reconnection with nature, including animals, needs to be founded in values that foster a new commitment to nature and the ecological environment [3]. In the discourses on life and death in African philosophy, it is visible that animals are viewed as creatures that are in a hierarchy below human beings, and accordingly they are often not valued or taken into consideration in research on life and death. Further, to end the life of an animal through killing seems to be acceptable, and moral bonds between humans and animals are rarely discussed in African philosophies when it comes to life and death. However, there seems to be a new trend in eco-philosophy and eco-psychology to shift the focus from an anthropocenic worldview to a more eco-entered worldview, which is urgently needed to expand socioecological ethics for environmental protection [3], particularly when leading discourses on wildlife (crime).

### 2.6. Meaning and Absurdity 

Meaning in life is a well-researched concept in general, as well as in existentialism [89,90,91,92,93,94,95], and often meaning-making has been associated with mental health and the ability to transform the “meaninglessness” of life and its absurdity into strengths to overcome challenges [96]. Camus pointed out that human existence is meaningless and that striving for meaning in a life and a world that does not have any inherent meaning is absurd [70]. Absurdity has often been discussed in connection with meaning, and meaning is connected to the natural world and the experience of natural phenomena [63]. A life without meaning is viewed as a life that does not matter [97] and that is without worthiness [96]. Steger [92] pointed out that meaning is created through an overall meaning system and that it has been highlighted earlier that the “essence of meaning is connection” [98,99], whereby the experience, relationship-building and cultivation of connections with nature contribute positively to the meaning-making process [76]. Additionally, studies have supported the idea that connectedness with nature contributes positively to meaning in life and well-being [100]. Nature presents itself as a circle of living and dying, representing birth and death [63], whereby the meaning of life should be of higher value than the meaning of death according to the philosopher Camus [96]. However, reflecting on meaning in life brings about the necessity to reflect upon the absurdity of life [96], as well as a discourse on ethics and the imprisonment of the mind through enslaving thought systems, which are often imposed through unconscious thoughts, feelings and behaviours [101]. In times of crises, un unaware mindset might lead to a certain urge to behave in a specific way, only seeing the choice between death and death while losing the connection to certain values, humanism, human connections, community and the joy of the beauty of nature [102]. Absurdism suggests that life brings about hardships and that humans need to accept this harsh reality, transform suffering and pain and keep on living while seeing the absurdity in life [103] Further, Camus [104] emphasises that the world is a hostile place; however, the sources of inhumanity are people themselves.

With regard to African philosophy, not much research has focused on meaning in life from African (philosophical) perspectives [105]. Here, recently four different approaches to meaning in life have been distinguished: the African God–purpose theory of meaning (highlighting that meaning is created through God’s purpose and destiny); the vital force theory of meaning (increasing meaning through a focus on what makes vital forces stronger); the communal normative function theory of meaning (meaning-making through harmonious interaction with one’s own community); the consolationist theory of meaning (striving for perfectionism in life as a meaning-making process) [105].

Meaning in life is ascribed to certain values, and according to Metz [106], meaning in life is particularly informed by two African philosophical traditions, namely community and vitality, whereby vitality theory is even more important in life than community theory, always connecting meaning rather to creating vitality rather than harmonious relationships with the community. Agada [107] also refers to the African vital force theory and highlights that a vital force theory that only focuses on a naturalistic perspective of the world as a whole and the universe leads to the idea of the meaninglessness of life from an African point of view. The author emphasises that this kind of perception of a meaningless life *per se* does not fit with African worldviews, which is why she points to a transcendalist framework of vitalism that takes God into consideration as an ultimate guarantor of meaning. As such, vitalism theory is combined with destiny theory, meaning the God–purpose theory. Mlungawana [108] points out that life, love and destiny, again relating to vitality theory in combination with a God-related purpose theory, create a meaningful life from and African perspective. Life might be viewed as a connection to what Metz [109] calls vitality, namely living one’s life actively and with a vital force. However, Mlungawana [108] points out that destiny is the most important theory related to creating a meaningful life from African philosophical traditions. In another article, the author discussed the concept of absurdity in African philosophical thought and pointed out that absurdism, as found in existentialism, is unacceptable in traditional African religion, which refers to the meaning of existence through a Supreme Being [110]. A recent article by Molefe [111] highlighted that the concept and conceptualisation of personhood in African philosophy is mostly important in constituting a meaningful life. As such, the person is viewed as a human being when behaving within the context of defined ethics of personhood. The ethics of personhood are mainly defined by the connection of moral excellence or perfection, which means that a meaningful life can only happen when a person strives and displays ethical and moral behaviour [110] (Table 2). 

### 2.7. Freedom and Determinism 

Western research has shown that individuals being exposed to nature experience an extended sense of freedom and autonomy [112,113]. Existentialism proposes that humans do have free will, leading to the idea that humans can create their own meaning in life [104] and that they are free of an external structure, being responsible for themselves [71]. They are further free to think and act, meaning they have to take responsibility for their actions [62]. Sartre, who is one of the most popular French existentialists, emphasises that individuals are born free, because they have the “freedom of definition of man”, referring to the freedom to distance oneself from the self-image and the other image. Freedom is at the same time bound to responsibility, meaning that each and every person is responsible for their own world and the choices they make in the world [114]. For Sartre, each and every individual has freedom of choice, which should include the individual’s responsibility and the freedom from identification from other individuals and from ego [115]. Each choice an individual takes is a choice of self-definition and one’s own identity [114]. Sartre’s concept of radical freedom and choice has been criticised and researchers have argued that there are determinizing factors that influence the choices of individuals, such as psychological factors, aggressive drives and environmental and historical factors [116]. This statement counteracts Sartre’s idea that there is no human nature as such, rather only radical freedom and choice, which lies in each and every daily decision. Sartre [62] further emphasises that there is no purpose in life except living one’s own freedom. This means that humans find themselves existing and then have to create themselves towards their essence, based on the one and only existing value of freedom [62]. Others have agreed that freedom of the mind is one of the most important values in counteracting the absurdity of an infinite death [117]. Several determinists, however, counterargue this freedom, highlighting that humans are bound to situational and contextual forces, as well as economic and productive forces, and that the past and principles of universal causality influence freedom and choices [118]. Sartre’s philosophy breeds hope, optimism and courage, since it presents human beings as most influential and free to do whatever pleases them. This is possible when consciousness arises, because being conscious provides the individual with the recognition of opportunity and choice, as well as a potential acceptance for being responsible for one’s own actions [62].

According to O’Donohoe [119], Sartre’s existentialism has “real consequences” for individuals and social groups and in defining how to live an authentic and meaningful life. However, the question remains—how is freedom conceptualised in African cultural contexts (Table 3)?

Basically, freedom is “the right and power to act or behave as one chooses. It is the absence of internal restraints and external constraints” [120]. Okaneme [121] points out that concepts of freedom in African perspectives differ fundamentally in the way that freedom in African philosophy is based on a collectivistic and communal worldview, while Western philosophies usually see it as part of the individual nature. Dukor [122] defines freedom in African perspectives as the freedom of harmonizing one’s actions with the norms and values of the society. The author explains further that from an African perspective, there is no possibility of absolute freedom, since Africa has been made dependent by Western colonialists, removing free will [122]. Other authors, such as Ezenwankwor [121], counteract Dukor [122], emphasizing that Africans, as human beings, do have the free will to either accept or reject colonialism as a predetermined event and can make a choice to accept or reject responsibility for one’s own actions. 

“To be morally responsible for one’s actions is to have the power to be the ultimate creator of one’s intentional acts or behaviour. Any lack of power to be one’s intentional creator of his own actions ultimately implies that the actor is not free and therefore not morally responsible” [121].

When Africans feel that they are not morally responsible for their own actions through the experience of the colonial legacy, according to Ezenwankwor [120] this means that they do not have free will and are limited in their choices and their responsibility. Why is this view strong in African contexts?

Freedom is always defined within the norms and values of a social group, which is why Africans usually foster a determinist point of view. Several authors have emphasised that dealing with the concept of freedom is a challenge in African contexts, since it is often seen as part of a philosophical and ideological struggle that is backed by a defense of traditional African thought [123]. However, freedom can also mean the free interplay of ideas, as well as the freedom from Western ideas and mindsets. Dukor [123] also points out that the freedom of humans is determined by the freedom God determines for humans. As such, self-consciousness, existentialism, materialism and spiritualism are all concepts that play a role in freedom in Africa. Finally, freedom is also to overcome categories that have been set in the past, such as racial categories [123]. The acceptance of pluralism and humanity means freedom of African thought [123]. According to the authors, there are other aspects that play into African viewpoints on what freedom means—it is reflected in the unity and identity of all humans, incorporating idealist, spiritual and materialistic conceptions of freedom.

Gwara [124] presents African perspectives on freedom as extremely complex and places freedom within the discourse on destiny, which also impacts strongly on meaning in life. The author argues that freedom is a concept that incorporates determinism and freedom at the same time: from an African perspective, life’s circumstances surrounding the individual’s life events are determined, while the individual’s will and actions are undetermined. Gwara [124] defines this view as soft-determinism. Mungwini [125] reflects, however, that the concept of determinism is critical with regard to self-determinism and self-liberation, emphasizing that African philosophy “has positioned itself at the centre of the struggle for justice and self-determination” [125], The struggle is placed within the emancipatory urge, African identity and context–orientation discourses, whereby African philosophy seems to hide behind abstract quests for knowledge without taking practical and ethical commitments into consideration [125].

### 2.8. Community and Aloneness 

Aloneness, also referred to as isolation in the existentialist literature [1,23,71], can easily become a source of meaninglessness in life [126]. However, it can also be seen as a “suffering towards” mental health and moral growth from McGraw’s [127] point of view and when the experience of it is used for developing sensitivity, refining, deepening and purifying. From an existential point of view, there are different ways of feeling alone, such as metaphysical loneliness (the experience of the separation of the self from the world), epistemological loneliness (the experience of a conscious loneliness that one cannot escape from), communicative loneliness (the experience of the inability to communicate effectively with others), ontological (intrapersonal) loneliness (a lack of self-intimacy and meaning within oneself), and ethical loneliness (loneliness inherent in freedom, choice, value and responsibility). Furthermore, there are existential loneliness (the experience of having to go through life’s stages on one’s own), emotional (eros) and social (friendship and community) loneliness, cultural loneliness (experience of being overlooked, unconnected, disconnected from mainstream societies) and cosmic loneliness (the impression of being all on one’s own in an impersonal, chaotic universe). According to McGraw [127], all forms of loneliness evoke the (re-)definition of the network of relationships and community. If a person is social and enclosed in the network of community and social relatedness, loneliness is shattered. Existential loneliness can be overcome by reframing one’s experiences of loneliness in life and by identifying new meaning in life [128]. Meaning can only be created within the context of the other and authentic living, according to Buber [129], is only possible through constructing serious relationships.

The concept of community and being part of a community is extremely important in African life and philosophy, as highlighted above [104,105,108,110,111]. Community is viewed as one of the crucial aspects of developing as a person and building an acceptable personhood that is sustainable and holistic [130]. As such, individuals as seen as social by nature and humans should develop cooperation and common goals and values to adhere to, while the community must produce what is good for the individual at the same time. While Mbiti [84] points out that non-human parts of creation are objects, Tutu [131] highlights that humans should be non-exploitative and treat all life on earth as God would. This is possible when the community is experienced at fostering creative, valuing and respectful relationships. This kind of supportive community can then support individuals to respect nature and all living beings on earth, as God would do. Tutu, as an archbishop by profession and calling, uses the God–purpose theory, highlighting that humans should care for the world in a holistic way, and as God wishes, in an interdependent and respectful way.

While there are surely different discourses on community in African thought, there are also discourses on non-existence—similarly to writings on existentialism, such as presented by Sartre [62]. However, it is emphasised that the discourse on existential non-existence seems to be contrary to African thought in general, which usually incorporates the idea that a (impersonalised) Supreme Power exists, which represents meaning as well as being [104]. According to Attoe [104], the metaphysical thought in African traditional religion and philosophy does not leave any space for nothingness, as explained by existentialists such as Sartre [62]; rather, it provides for the idea of a differentiation of “being and being-alone” [104]. Since God is viewed as eternal, there is no room for nothingness; even if the universe does not exist, there can only be aloneness, but not nothingness. Therefore, in African thought, the idea is that there is only the concept of being in two ways: as “being alone or being with others” [104]. However, the aloneness of individuals can never be a nothingness, since even in the concept of being alone, the Supreme Being exists at the top of the hierarchy of beings [131,132,133]. What makes life and living meaningful is rather the vital force that contributes to an increased quality of life, which is also what differentiates human beings from animals—the vital force, which is infused by spirituality and which thereby transcends animal nature [104]. This vital force seems to be stronger in community than in aloneness or isolation and grows with the expression of creative power, productivity and moral obligations, which have impacts on others, and at best on the community [104]. The communal foundation of thought is extremely important in African philosophy and has been viewed as a priority in African philosophy, since it supports the understanding of reality and the implementation of socioethics in the community [105].

Wildlife is extremely important for many African communities, and many ethnic groups in Southern Africa are strongly related to wildlife through their culture and heritage values [134]. Wildlife and community are strongly connected through existential values, whereby the mere existence of wildlife provides value for the people who have lived in close interrelationships with wildlife from many centuries [135]. However, in many parts of Africa, humans are in conflictual interrelationships with natural systems and wildlife, especially where there is competition for land or other resources. In such cases, the competition between humans and wildlife interferes with constructive, meaningful, purposeful, God-led and holistic interactions [136]. Several community-based approaches to managing natural resources and wildlife have been implemented in Southern Africa; however, constructive community–wildlife and natural-resource-based conservationist interactions can only be implemented when communities feel that they gain benefits from it—which is often not the case [137]. This perspective is often not the one expressed and favoured by Tutu [131], who rejects the God-led purpose of valuing and respecting nature as such and rather embraces the perception that nature and non-human creatures are viewed as objects rather than as part of a holistic interaction at the subject level (Mbiti, 1990). It further shows that the traditional hierarchy of beings is existent in African thought, which places animals and plants below human beings [132,133,138], meaning they might not be treated on an equal, respected and valued level (Table 4).

## 3. Conclusions

This theoretical article explores (eco-)existential perspectives, Greening’s [1] four givens and selected African philosophical concepts, aiming to understand the complexities behind the prevalence of wildlife crime and the impossibility of effectively combatting wildlife crime within global and African contexts.

### 3.1. Life and Death

The investigation into psychological and philosophical approaches shows that wildlife crime might be accepted to a certain degree in African contexts, since animals are seen as lower in hierarchical status in comparison to humans, and since the focus of humans seems to be primarily anthropocentric. As the movement of eco-psychology or eco-philosophy is a recent one, it needs to be expected that individuals will only counteract wildlife crime actively when they have changed their mindset towards one that values animals and wildlife on a similar hierarchical level and from an ecocentric perspective.

### 3.2. Meaning and Absurdity

Usually in the (eco-)existentialist literature, meaning-making is strongly connected to the individual, their values and their approach to life. Positive and constructive actions (as proposed by Camus) are important in creating meaning, as are nature and natural experiences. Through wildlife crime, individuals connect to nature, although in a destructive, domineering and non-ethical way rather than in a constructive and positive way. Meaning creation in African thought—as with the concepts of life—is focused on the individual as part of the bigger system, either connected to God’s purpose and life as such (vitality) or the interaction with one’s community and perfectionism (to become a person through other persons). Meaning-making is hardly connected to natural experiences, e.g., positive interactions with the natural environment, plants, animals or wildlife. Again, meaning-making is strongly determined by the individual’s connection to either a Supreme Being or one’s community. Meaning-making is not related to connections to other African objects, which often might be viewed as being lower in the natural hierarchies. 

### 3.3. Freedom and Determinism

Since existential voices call for individuals to be conscious and aware of their own ability to be free and liberate themselves from limiting ideas and behavioural patterns, African philosophers are still very much anchored in the view that the individual, as well as the group, is determined by external and internal factors, which are connected to the destiny that is created by God or a Supreme Being. Africans can never be fully free due to colonialism and historical events, as well as God’s impact in their life. This means, that individuals and social groups might not take responsibility for unethical actions, such as wildlife crime. They might not feel free to act against the killings of animals, and instead might ascribe it to the destiny of the wildlife criminals on the one hand and to the destiny of the world and the animals, implemented by God, on the other hand. In African philosophies, there are only very soft voices that call for responsibility to be taken for thoughts, ideas and actions and for choice, freedom and new opportunities. This deterministic attitude might be fatal when it comes to wildlife crime, since humans will not take responsibility and protect animals and wildlife, in particular when animals are viewed as lower in the hierarchies of the natural environment than humans.

### 3.4. Community and Aloneness or Isolation

The anthropocentric view proposes that community is of very high value in African contexts and that individuals can only grow into becoming a person when involved in a community. Isolation and aloneness might easily lead to disrespect of others, but also towards disrespect of nature, animals and wildlife. If wildlife crime is to be stopped, this will only happen through the efforts of communities in the Southern African context. For communities, value needs to shift from the anthropocentric view towards an ecocentric view of human–animal or human–wildlife relationships.

### 3.5. Concluding Remarks and Recommendations

In conclusion, neither the eco-existential approach to relationships between humans and nature, animals and wildlife nor the African philosophical constructs explored seem to be strong enough to influence value orientations and evoke stronger counteraction at the international level against wildlife crime. Both existential and African discourses seem more anthropocentric than ecocentric, and are still far away from developing an ecocentric perspective.

Wildlife crime can be combatted through the implementation of various policies and the development of forensic and investigative tools, crime science practices and anti-poaching and anti-crime training. To combat wildlife crime, radical shifts need to occur in conscious thought and mindset in order to move existential–anthropocentric positions towards eco-existential positions in Western and African mindsets and towards ecocentric approaches and values. Conscious decisions must be made on how to define life and death, meaningfulness and absurdity, freedom and determinism and community and aloneness or isolation in order to make radical changes related to human–animal and human–wildlife interactions. Humans need to include death into their own perspectives in order to stop killing animals, thereby externalizing death within creatures that are viewed as marginalised objects in the natural realm. Humans need to create a mindset focused on leading meaningful, vital lives, accepting certain degrees of freedom of choice, decision-making and responsibility. The decision to protect animals and nature in human–wildlife interactions requires the formation of a psychological and philosophical concept of the world that goes beyond humankind and one’s own community, as well as the will to strive for growth that is defined by ethical and moral behaviour rather than anthropocentric meaning, vitality, community and perfectionism.

This article presents a limited view and only investigates selected parts of the complex philosophical discourses from different social, historical, cultural, historical and economic backgrounds. This represents a first attempt to bring Western existential and African philosophical thoughts together and to explore and investigate the shifts in thought and mindset needed at psychological and philosophical levels to combat wildlife crime in an impactful way.

## Figures and Tables

**Table 1:** Life and death.

Existential Givens: Life and Death	Selected African Concepts	Wildlife Crime
Life is a path to death. Authentic living is only possible when one confronts death.	God is the creator of lilfe and death is just an entry into another world where the soul lives on. An individual lives to become a person and promote human flourishing.	Eco-existential perspectives call for the reconnection with nature, the protection of nature and animals. African approaches are primarily anthropocentic and focus on the life of humans within their community. The life of animals from African perspectives may be exploited since animals are viewed as being of lower hierarchy and status in the world of the living. Latest African philosophies also call for an eco-philosophical approach.
Death is a natural process and being consicous about death can help in living a meaningful and authentic life.	Death is a calling from God and a transition into another realm. It is a communitarian event.	Both views do not accept human killing and suicide. Very few literature focuses on the killing of animals. While it is assumed that a slow and expected death is better for the transition of a human, there does not seem to be a discourse on the transition of animal souls from life to death in African philosophies.

**Table 2:** Meaning and absurdity.

Existential Givens: Meaning and Absurdity	Selected African Concepts	Wildlife Crime
Meaning-making is important and meaning can be created through humans.Meaning can be created through natural experiences.Connection creates meaning.The sources of inhumanity are the people themselves.	Meaning is created in four ways: - God’s purpose and destiny creates meaning - Strengthening vital forces creates meaning - Meaning-making through harmonious interaction within one’s community - Meaning through striving for perfectionism The life is never meaningless per se. Personhood is an overall topic in meaning of life creation through ethical and moral behaviour.	Eco-existential perspective need to find a broader acceptance and consciousness must be build with regard to the meaningfulness of natural experiences and positive human/nature/animal/wildlife interactions to combat wildlife crime.In terms of African approaches to meaningfulness, the anthropocentric worldview needs to be overcome and transformed into a more ecocentric perspective in which humans and animals are both valued on a similar level and where meaningfulness concepts might not only focus on human-human, but also on human/nature/animal/wildlife interactions.
Existence is absurd and brings hardships, which need to be accepted and transformed.	There is no indication of the concept of absurdity in African philosophical concepts.	

**Table 3:** Freedom and determinism.

Existential Givens: Freedom and Determinism	Selected African Concepts	Wildlife Crime
Freedom: Humans have free will, can create their own meaning, are responsible for their actions since there is freedom of choice. Purpose in life is to live one’s own freedom.	There is no complete freedom for Africans due to the world’s history and God’s destiny for the individual. Therefore, ethical and practical commitments are not taken and accepted. Responsibility of the individual and the group is rejected.	Humans need to become aware of their own freedom and their responsibilities in the world and should not view themselves as limited by other factors other then themselves. Then they cannot justify unethical behaviour anymore: If wildlife crime can still be justified through poverty and colonialist structures, it cannot be combatted effectively.
Determinism: People are determined by the circumstances and their limiting (external and internal) factors	African philosophers are determined by colleactive and communal values and worldviews and philosophers do believe that due to eternal and internal (historical) factors, African can never be free. That means that the mindset is limited by factors which are in the past and out of control. Life events are seen as determined.	

**Table 4:** Community and aloneness or isolation.

Existential Givens: Community and Aloneness/Isolation	Selected African Concepts	Wildlife Crime
Community helps to create meaning and an authentic life, in particular when serious relationships are created.	Community is extremely important in African philosophical concepts, since through the experience of interactions and community, an individual can grow into a person. Only when developing within a community, can a person develop respect for nature and other creatures. Moral obligations can only be experienced and lived when growing up and living in community.	Nature, animals and wildlife are often not respected and valued as such, since communities feel in conflict over resources with animals/wildlife, in particular conflict over land. Communities are values while animals/wildlife are rather seen as objects than as living creatures, although there is a small movement that emphasises that nature and animals/wildlife need to be respected as such.
Isolation leads to a meaningless life, however different concepts of aloneness or loneliness can lead to individual growth.	Isolation, loneliness and aloneness create disconnection and disrespect for other humans and for natural creatures.	

## Data Availability

Data are available in the public domain.
